# The Face of Emotion: Botulinum Toxin, Emotional Anatomy, and Mood Modulation

**DOI:** 10.1111/jocd.70264

**Published:** 2025-05-27

**Authors:** Robert J. Vanaria, Aysham Chaudry, Angelica C. Marrero‐Perez, Z. Paul Lorenc, Mark S. Nestor

**Affiliations:** ^1^ Center for Clinical and Cosmetic Research Aventura Florida USA; ^2^ Hackensack Meridian School of Medicine Nutley New Jersey USA; ^3^ Lorenc Aesthetic Plastic Surgery Center New York New York USA; ^4^ Department of Dermatology and Cutaneous Surgery University of Miami Miller School of Medicine Miami Miami Florida USA; ^5^ Department of Surgery, Division of Plastic Surgery University of Miami Miller School of Medicine Miami Miami Florida USA

**Keywords:** BoNT‐A, botulinum toxin, emotional mimicry, facial feedback hypothesis, mood regulation, neuromodulators, wellbeing

## Abstract

**Background:**

Facial expressions profoundly influence emotional communication and well‐being. The facial feedback hypothesis suggests that facial muscle activity can reinforce or modulate emotional experiences. Botulinum toxin type A (BoNT‐A), known for its aesthetic applications, is emerging as a modulator of mood through its effects on facial musculature and feedback mechanisms. Its ability to influence mood extends beyond aesthetic improvement, offering potential therapeutic benefits in emotional regulation.

**Aims:**

This literature review explores the interplay between facial anatomy, emotional expression, and the potential of BoNT‐A to enhance overall mood and well‐being, beyond aesthetic improvement.

**Methods:**

A literature review was conducted using PubMed with search terms like “botulinum toxin” AND “emotion.” Relevant English‐language articles from 2000–present were selected based on their examination of BoNT‐A's influence on mood and facial feedback. Articles not in English and lacking relevance were excluded. Citation tracking was used to identify additional studies, and insights from the authors' clinical expertise further informed the review.

**Results:**

A total of 46 articles were included based on their relevance to both BoNT‐A and emotional states. BoNT‐A‐induced muscle relaxation can prevent specific muscle contraction and thereby disrupt feedback loops that reinforce mood, potentially enhancing positive emotions and promoting overall wellbeing. Studies suggest that BoNT‐A injected into the glabella region can modulate amygdala activity, reduce symptoms of depression and anxiety, and strengthen overall emotional resilience. By modulating facial expressions, BoNT‐A can also enhance social interactions, increase positive emotional contagion, and contribute to a more positive self‐perception.

**Conclusion:**

BoNT‐A shows promise as a novel approach to mood regulation and enhancement by altering facial feedback mechanisms. Its dual aesthetic and therapeutic benefits highlight its value in both dermatology and mental well‐being.

## 1 Introduction

Human evolution has produced the ability to read others' emotions via facial expression even if they do not outwardly say what they are feeling or even say otherwise. The small muscles of the face respond to the limbic system's output by contracting and relaxing in synchrony to produce the faces associated with emotions, like smiling when happy and frowning when sad. Smiling and other positive facial expressions contribute to reinforcing and maintaining positive emotional states. Repeated contraction of the orbicularis muscles, often activated when making a smile, results in lateral canthal lines, often referred to as “crow's feet.” Conversely, when frowning, the glabella (the corrugator muscle group and procerus muscle) contracts, furrowing the eyebrows inferomedially and conveying negative emotions such as anger, fear, and sadness. These emotions are also often linked to depressive mood states. Interestingly, Darwin referred to the corrugator muscles as “grief muscles” due to their association with sadness and mourning [1, 2]. Along with the medial frontalis muscle, excessive, chronic frowning can form an omega‐shaped wrinkle pattern termed the “omega sign” (omega melancholicum), frequently observed in individuals with depression and other mental health conditions [2]. Studies have shown marked overactivity of the corrugator muscles in depressed patients [2, 3]. The musculature of the face responds to emotional changes with a unique speed and sensitivity and the facial expressions associated with happiness, sadness, anger, fear, surprise, and disgust are believed to be universal. Research indicates that facial expressions are key indicators of an individual's psychomotor state [4].

Botulinum toxin has been a significant player in aesthetic enhancement since the early 2000s [5]. This bacterial isolate has demonstrated exceptional use in the aesthetic world for paralysis of the muscles that cause wrinkle lines to appear. Today, it stands as one of the most popular aesthetic treatments, with over 4.7 million procedures performed in 2023 alone [6]. It induces temporary flaccid muscle paralysis, affecting both voluntary and involuntary muscle functions [7]. Experimental clinical evidence has shown that facial botulinum toxin type A (BoNT‐A) injections can modulate the perception and processing of emotional stimuli, including the activation of the amygdala, an important structure in the limbic system involved in processing emotions [8, 9, 10, 11]. This is referred to as the “facial feedback hypothesis,” and it posits that facial expressions can influence emotional experiences through proprioceptive signals reinforcing these expressions [12]. That is to say, rather than beginning in the limbic system and displaying on the face, emotions can be reactive and experienced by patients by contracting the facial muscles associated with the corresponding mood state [12].

According to the facial feedback hypothesis, paralysis of the facial muscles with BoNT‐A may significantly influence mood expression. For example, injecting BoNT‐A in the glabellar region would interrupt negative emotional feedback loops, lessening negative emotional states while enhancing positive ones. Beyond simply reducing negative affect, BoNT‐A treatment may foster an increase in positive emotional expression, leading to greater ease in social interactions and heightened overall well‐being. BoNT‐A is known for its aesthetic benefits, but it can also improve social interactions and reduce irritability, anxiety, and depression. This literature review aims to evaluate the interplay between emotional expression and facial anatomy, along with assessing the use of BoNT‐A on mood changes using physical anatomy data and its potential for enhancing overall mood and well‐being in patients.

## 2 Methods

A PubMed search was conducted using the terms “botulinum toxin” AND “emotion.” Results were screened to include English‐only articles between 2018 and 2024 and excluded duplicates. Further exploration of BoNT‐A's emotional effects was enhanced through citation tracking, additional PubMed searches, and authors' clinical expertise. A flowchart is included for methodology tracking.

## 3 Results

**FIGURE 1:**
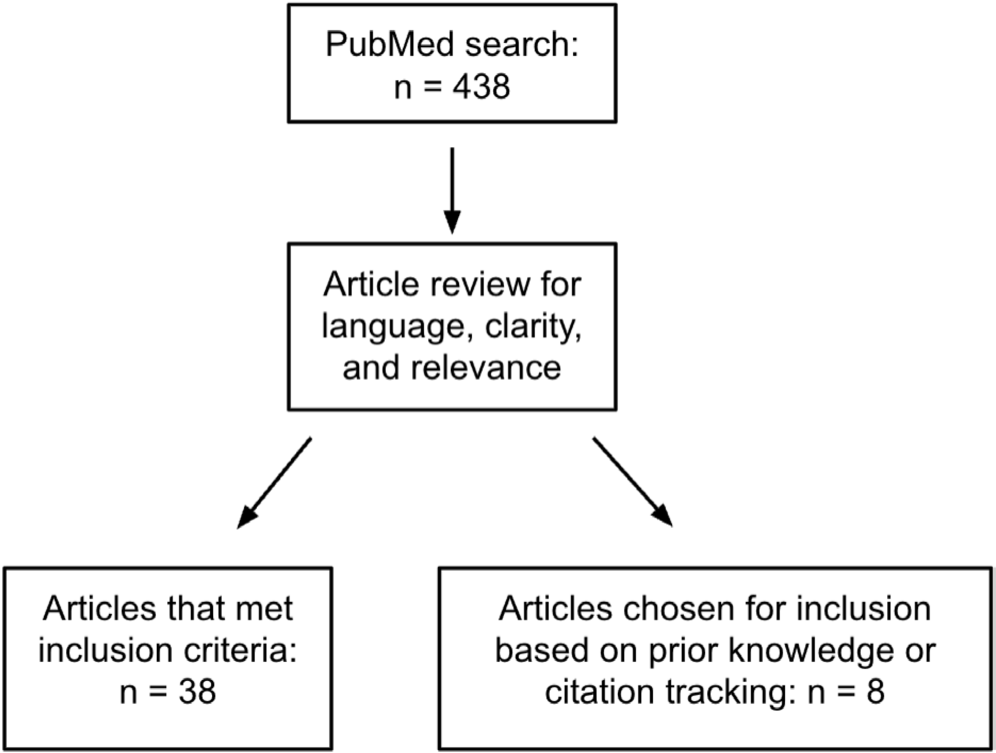
Flowchart depicting PubMed search strategy and article inclusion.

A total of 46 articles were included based on their relevance to both BoNT‐A and emotional states. Results were categorized into three different categories based on their relevance to different topics related to this review: facial anatomy and emotion, emotional sequelae, and social and interpersonal effects. Inclusion in one of the categories did not preclude data inclusion in another. Potential limitations to our results include inclusion of author expertise and citation tracking, which may result in selection bias. A flowchart is included below illustrating the search process for articles included in our literature review Figure 1.

## 4 Discussion

### 4.1 Facial Anatomy and Emotion

**FIGURE 2:**
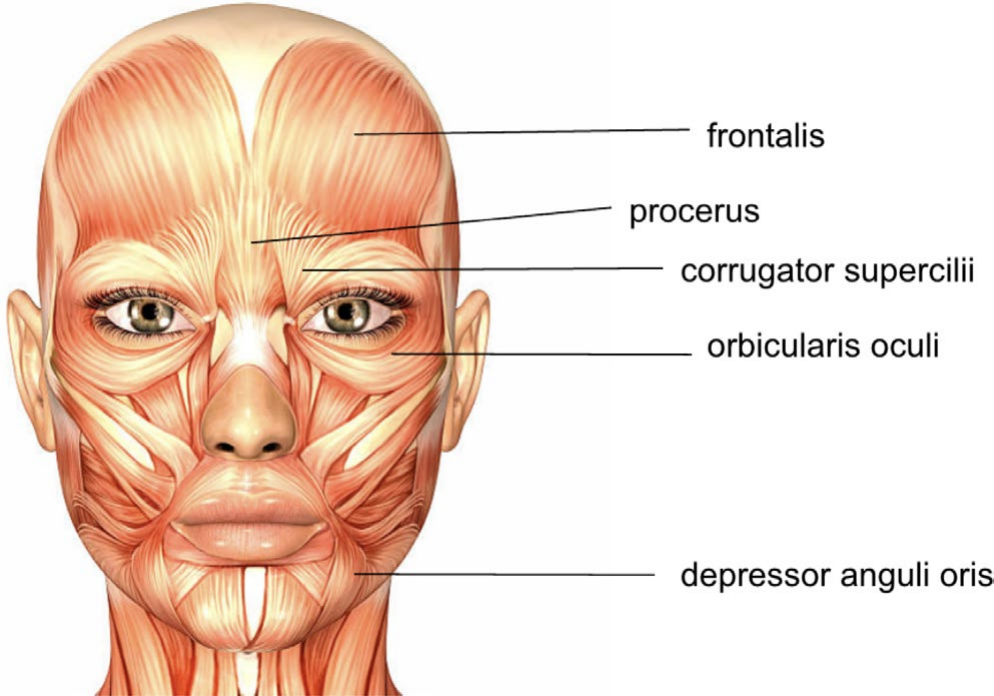
Facial musculature anatomy including glabellar complex.

The frown is formed by the glabella, consisting of muscles that contract to depress the medial eyebrow. These muscles are depicted in Figure 2. The furrow lines or wrinkles created by the chronic contraction of these muscles are referred to as glabellar lines, frown lines, or angry elevens. Contraction of the corrugator supercilii and procerus are principally responsible for the display of negative emotions, such as fear, sadness, and anger [5]. Because the contraction of these muscles displays emotions such as anger, fear, and sadness, the lines formed by the muscles become more prominent with additional muscle contraction, for example prolonged or frequent frowning [6, 7].

The smile is a more complex, coordinated effort of facial muscle contraction. The “true” or “Duchenne” smile is often referred to as a genuine smile and involves contraction of both upper and lower facial muscles, specifically the muscles involving the mouth and those encircling the eyes: zygomaticus major and orbicularis oculi [13, 14]. This smile consists of both showing teeth and squinting the eyes. The muscles that produce this smile are ultimately responsible for the display of emotions such as happiness, joy, and friendliness. Smiling plays a crucial role in reinforcing positive emotions, and the facial feedback effect from smiling is stronger when orbicularis oculi muscles are contracted [4]. However, similar to frown line formation, the contraction of the orbicularis oculi results in lateral canthal lines, often referred to as “crow's feet,” which are a common treatment area for BoNT‐A. The ability to form and express a genuine smile is integral to social connection and emotional well‐being, and its preservation is an important consideration in facial aesthetic treatments.

Producing a facial expression is perceived by the brain, contributing to the feeling of emotion. Researcher Fritz Strack highlighted the importance of the relationship between facial expression and the brain through his research on the “facial feedback hypothesis.” Results from his studies indicated that people rated cartoons as funnier when they held a pen with their teeth compared to holding it between their lips [15]. He interpreted this intriguing observation by suggesting that the positioning of facial muscles affects an individual's subjective enjoyment of the cartoons: holding a pen between the front teeth mimics the facial expression related to smiling, while positioning it between the lips engages muscles, such as the orbicularis oris, that do not promote smiling. This effect illustrates a bidirectional connection where perception can influence actions, which in turn feed back to alter perception [15]. Similarly, Larsen et al. found that participants who held two golf tees in the frown area and were told to either touch the tips together or keep them separated rated negative images as more unpleasant when touching them together than when holding them apart, which supports the facial feedback hypothesis [16].

The embodied emotion theory states that physiologic feedback from facial muscle contraction creates a feedback loop within the brain that reinforces that emotion (positive feelings when smiling and negative emotions when frowning) [14]. External expression of emotions enhances the internal experiences of those feelings.

Research has long indicated that feedback from facial muscle activity during expression is crucial for experiencing emotions. Importantly, a number of psychological clinical studies have shown support for the facial feedback hypothesis [14, 15, 16, 17, 18, 19, 20]. Researchers have utilized facial electromyography (EMG) and demonstrated that the activity of the corrugator muscle is heightened when observing images of angry faces [21, 22, 23]. This increase in corrugator activity mirrors reactions to negative emotional images and sounds [16, 24, 25, 26]. This conglomerate of data suggests that tailored interventions targeting and subsequently paralyzing specific facial muscles with BoNT‐A can be used to modulate emotions and feelings.

### 4.2 Emotional Effects of Botulinum Toxin Treatments

BoNT‐A is approved by the Food and Drug Administration (FDA) for use in dermatology and aesthetics. Aesthetically, this neurotoxin effectively diminishes the appearance of wrinkles by inducing muscle relaxation, which in turn smoothens the overlying dermis [27]. Additionally, BoNT‐A has gained recognition in various medical specialties over time. Recent studies have yielded preliminary findings that indicate its expanded potential applicability as a mood modulator [28, 29, 30, 31, 32, 33].

Mood alterations can have profound impacts on patients across all fields of medicine, highlighting an important area for improved treatment modalities. Mood modulation as a result of BoNT‐A treatments has shown promising results. When treating the glabellar region specifically, BoNT‐A was found to lessen feelings associated with sadness and depression by resisting contraction of muscles associated with negative expression. This in turn has an uplifting effect on mood and can revert an altered mood state back to the patient's baseline [29, 31, 32, 33].

BoNT‐A treatment of the glabellar complex is able to make patients feel less angry, fearful, and sad due to the attenuation of negative emotion processing. After BoNT‐A treatment of the glabellar complex, patients are more likely to express positive facial emotions and have better emotional experiences [33]. A potential confounder for this observation could be the self‐esteem boost from aesthetic satisfaction that is achieved by BoNT‐A treatments and that muscle paralysis does not necessarily cause emotional modulation. However, multiple studies have shown that BoNT‐A treatments are associated with significant benefit in reducing anxious, depressive, and irritable moods, including meta analyses and in studies with patients with depression who failed multiple medications [6, 34].

Since the initial case series of Finzi and Wasserman demonstrated the antidepressant effect of BoNT‐A for major depressive disorder (MDD), multiple randomized control trials have confirmed that glabellar BoNT‐A is a strong antidepressant [6, 17, 18, 19, 20, 35, 36, 37, 38, 39, 40, 41]. Recently, glabellar BoNT‐A, but not periocular BoNT‐A, was shown to be effective for treatment‐resistant MDD, as predicted by the facial feedback hypothesis [41]. The MDD meta‐analyses have consistently reported a 45%–55% reduction in depressive symptoms, a 50%–60% response rate, and approximately one‐third of patients achieving remission after BoNT‐A injections [42, 43, 44]. These conclusions are supported by the large effect sizes reported in these meta‐analyses (e.g., Hedge's *g* = −0.82 or Cohen's *d* = 0.98) [42, 43]. Glabellar BoNT‐A has also shown efficacy for bipolar depression, social anxiety disorder, obsessive compulsive disorder, and generalized anxiety [45, 46, 47, 48]. The mechanism of action is thought to be through modulation of amygdala activity. Functional MRI studies have shown that glabellar BoNT‐A injection reduces amygdala activity [30, 31].

Compounding this is the fact that emotions are less well‐recognized in older (or older‐appearing) faces, and there are several reasons why this might be. Wrinkles and folds that are treated by BoNT‐A injections reduce facial decoding accuracy by adding more noise to the perceiver. Specifically, some wrinkles and folds resemble emotional expressions and can influence interpretation [49]. There is also data indicating that people pay more attention to same‐age faces, which in turn reduces emotion recognition accuracy across age groups (or perceived age groups in the presence of increased wrinkles and folds) [50]. Additional studies have shown that adding wrinkles and folds to avatar faces changed the interpretation of facial expressions from neutral to negative [51]. BoNT‐A therefore serves to not only decrease the experience and expression of negative emotions but can avoid unwarranted bias towards negative emotion interpretations by recipients. This is accomplished by reducing the presence and severity of facial wrinkles.

The facial feedback hypothesis is the impetus for the thought that facial expressions can influence mood. Many emotions have been shown to be linked to facial expression feedback, including enhancing feelings of enjoyment when producing a smile or disgust when wrinkling the nose and frowning [14, 16]. Treatments like BoNT‐A, which restrict an individual's ability to create a smile, can reduce the emotional feedback associated with smiling. Consequently, a person's mood could suffer if they receive treatment that limits the movement of the orbicularis oculi muscles, such as BoNT‐A for the treatment of lateral canthal lines (crow's feet lines), making them unable to produce a Duchenne smile and feel less happy when they smile. This has potentially substantial effects, as two studies have shown that patients who were making a Duchenne smile in their yearbook photo were less likely to get divorced and more likely to live longer than those who did not [18, 19]. It is essential to consider this point, as certain BoNT‐A applications (i.e., the treatment of crow's feet lines) may reduce wrinkles, but at the cost of diminished emotional experience.

Randomized control trials have shown that empathy is tied to facial feedback and the ability of the observer to read and react physically to stimuli, which can be measured by the “Reading the Mind in the Eyes” task [52]. By reducing the ability to form a physical emotional reaction, BoNT‐A treatments were shown to decrease patients' abilities to read others' emotions and to be empathetic [14, 53]. The emotional benefits of BoNT‐A can be maximized by carefully considering the treatment areas. BoNT‐A is effective for treating crow's feet, a popular patient treatment choice in addition to glabellar line treatment. However, as mentioned previously, this would limit the ability to form a full Duchenne smile. A more strategic approach would involve prioritizing treatment of the glabellar region and perioral muscles to both reduce expression of negative emotions via glabellar complex inhibition and enhance expression of positive emotions by avoiding treatment of the orbicularis oculi muscles to preserve the ability to form a Duchenne smile. By selectively targeting specific facial muscles, clinicians can optimize the emotional benefits of BoNT‐A treatment.

Davis et al. explored the facial feedback hypothesis through a comparative study involving subjects receiving either BoNT‐A or hyaluronic acid (HA) filler injections to assess BoNT‐A as a mood modulator. The BoNT‐A group was injected in the glabellar region and around the eyes, and they exhibited a significant reduction in emotional intensity, particularly for positive emotions/positive stimuli, when compared to the group injected with HA in nasolabial folds [32]. This effect both dampens the effects of emotional experience via the facial feedback hypothesis and also potentially impairs nonverbal communication. Similarly, a study from 2009 using functional MRI revealed that frown muscle paralysis decreases the activity of the amygdala and its neural connection with the automatic brain stem [54]. Similar results were replicated in 2014 [31]. The results indicate that facial feedback affects emotional intensity in specific situations, offering valuable insights for future research in emotional expression.

### 4.3 Social and Interpersonal Effects of Facial Expressions

**FIGURE 3:**
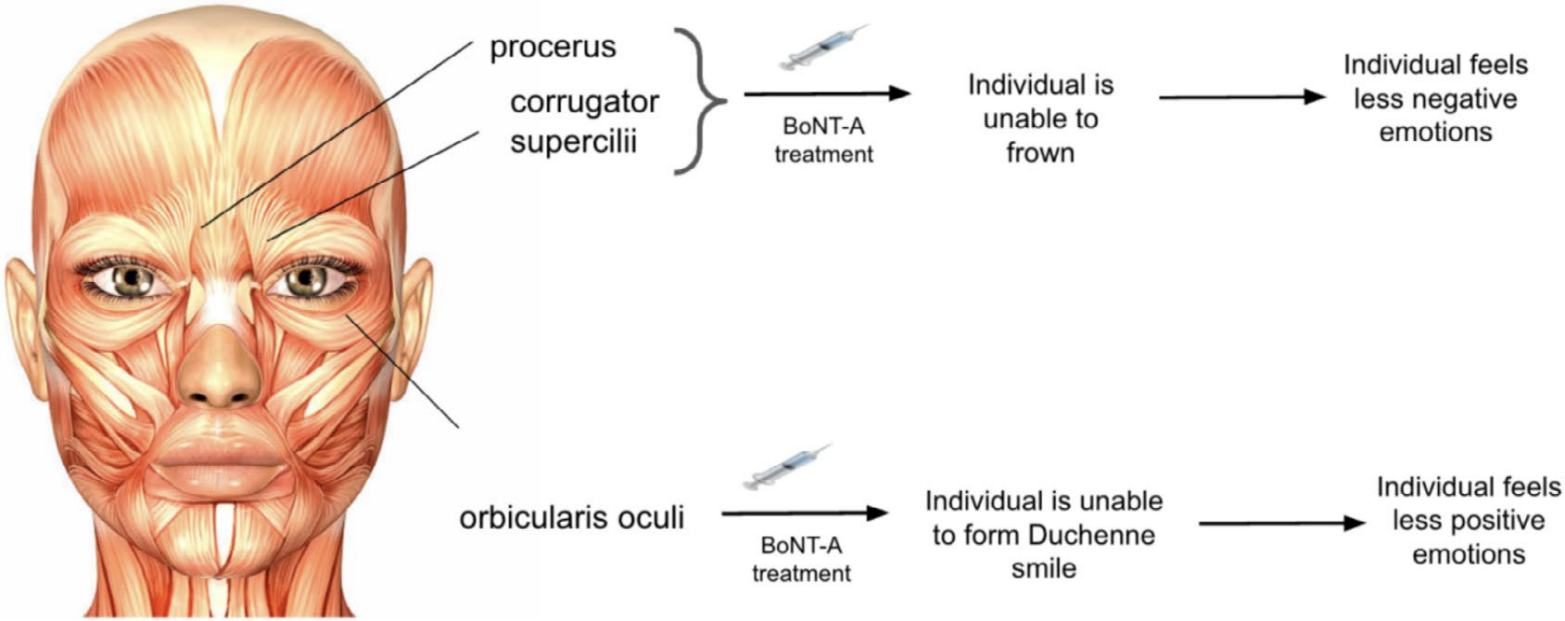
Schematic illustrating potential emotional outcomes of specific muscle‐targeted treatments.

Emotional feelings and expressions are both personal and interpersonal. We often project our internal emotions outwardly to those around us, while the emotional expressions of others can trigger responses within us. These dynamics are central to our daily lives, particularly in communication [55]. Emotional contagion refers to the observation that people tend to mimic and therefore experience the same emotions as those around them. It goes hand‐in‐hand with emotional mimicry [56, 57]. By combining the two theories of facial mimicry and emotion contagion, BoNT‐A‐treated patients may not only reduce the spread of negative emotion, but in turn spread positive emotion to people around them [54]. BoNT‐A effectively reduces both frown and lateral canthal lines, depending on injection location and the facial muscle being targeted. The treatment of these lines limits the ability to frown or form a Duchenne smile, respectively. This would also thereby change the emotional perception of the patient by the observer, particularly in settings where facial expression interpretation plays a larger role, such as loud settings or when part of the face is covered (i.e., by a mask) [24]. Figure 3 below illustrates the change in emotional expression dependent on which muscle is targeted with BoNT‐A.

**FIGURE 4:**
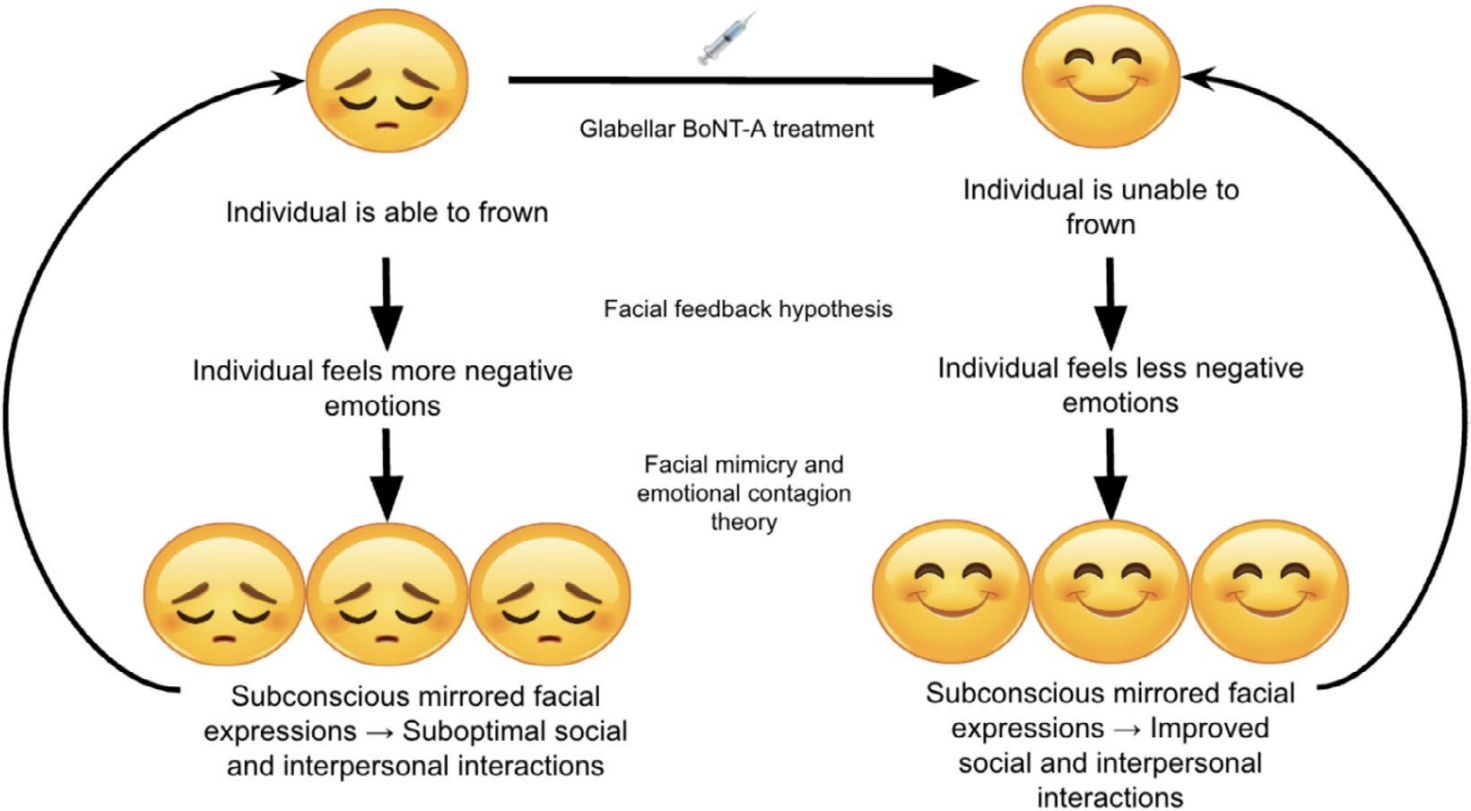
Visual representation of the compounding cycle of effects of BoNT‐A treatments with respect to facial feedback, facial mimicry, and emotional contagion.

Research suggests that BoNT‐A may be able to enhance positive social interactions in the correct context. For instance, by relaxing the muscles that contribute to frowning, BoNT‐A can potentially facilitate a more positive baseline facial expression, making individuals appear more approachable and friendly, which has positive social and professional implications. Furthermore, some studies indicate that BoNT‐A injections in the lower face, specifically targeting perioral muscles, may improve the expressiveness and emotional impact of smiling [58, 59]. This can lead to increased feelings of confidence and self‐esteem for social connection, as genuine smiles are more readily perceived and reciprocated by others [17, 60]. As a result, BoNT‐A injections would significantly affect interpersonal interactions and relationships. Figure 4 below demonstrates a visual representation of this theory. As an example, if a salesperson encounters a frown from a prospective client during a sales call, this may indicate dissatisfaction with the presentation. Consequently, the negative feedback from the prospect's facial expression might prompt the salesperson to respond with a frown as well, diminishing their chances of closing the sale. Thus, BoNT‐A injection can enhance our interactions in both personal and professional settings by making patients appear more positive and approachable to others. These traits are essential for individuals seeking productivity and success, and for cultivating positive relationships whether personal, romantic, or professional. The contagious nature of facial expressions amplifies the ability of BoNT‐A to induce positive emotions, extending its influence further as a mood modulator. These considerations are particularly important when we evaluate the implications and effects of BoNT‐A on emotional expression and communication, especially when deciding which muscles to treat with BoNT‐A based on patient request.

## 5 Conclusion

The relationship between emotional expression, facial musculature, and mood regulation presents a new potential for BoNT‐A treatments. The interaction between facial expressions and emotional experience supports the idea that facial expressions can influence mood states and perceptions of emotions. BoNT‐A, traditionally known for its aesthetic applications, shows promise as a modulator of mood by interfering with the feedback loops that reinforce emotions. By selectively targeting muscles responsible for emotional expression, such as the glabellar region, BoNT‐A can improve baseline mood and emotional states in patients. This leads to an improvement in overall wellbeing by patients. Incorporating mood‐enhancing benefits into aesthetic treatments can therefore expand dermatologic practice by addressing both physical and emotional wellness. As demand for holistic approaches rises, dermatologists have a unique opportunity through BoNT‐A to integrate mental health support within their aesthetic procedures, promoting a more comprehensive patient care approach while improving patients' overall wellbeing. Potential limitations to our study included search strategies that were non‐exhaustive of current literature. Future studies should attempt to enhance the quality of data regarding timelines for mood modulation and emotional benefit as well as establish more standardized guidelines and specific protocols for use in more specific patient scenarios with the primary goal of improving mood states.

## Author Contributions

All authors listed on this manuscript can attest that they have contributed meaningfully to its completion.

## Disclosure

The authors have nothing to report.

## Ethics Statement

The authors have nothing to report.

## Conflicts of Interest

The authors declare no conflicts of interest.

## Data Availability

The authors have nothing to report.
